# Identification of non-classical hCA XII inhibitors using combination of computational approaches for drug design and discovery

**DOI:** 10.1038/s41598-021-94809-x

**Published:** 2021-07-30

**Authors:** Mohammad M. Al-Sanea, Garri Chilingaryan, Narek Abelyan, Grigor Arakelov, Harutyun Sahakyan, Vahram G. Arakelov, Karen Nazaryan, Shaimaa Hussein, Gharam M. Alazmi, Haifa E. Alsharari, Waad M. Al-faraj, Faten S. Alruwaili, Nouf Q. Albilasi, Tahani S. Alsharari, Abdulaziz A. S. Alsaleh, Turki M. Alazmi, Atiah H. Almalki, Nasser H. Alotaibi, Mohamed A. Abdelgawad

**Affiliations:** 1 Pharmaceutical Chemistry Department, College of Pharmacy, Jouf University, Sakaka, 72341 Aljouf Saudi Arabia; 2 Institute of Molecular Biology of NAS RA, 0014 Yerevan, Armenia; 3 Institute of Biomedicine and Pharmacy, Russian-Armenian University, 0051 Yerevan, Armenia; 4 Foundation for Armenian Science and Technology, 0033 Yerevan, Armenia; 5 Department of Pharmacology, College of Pharmacy, Jouf University, Sakaka, 72341 Aljouf Saudi Arabia; 6 Department of Clinical Pharmacy, College of Pharmacy, Jouf University, Sakaka, 72341 Aljouf Saudi Arabia; 7 Department of Pharmaceutical Chemistry, College of Pharmacy, Taif University, P.O. Box 11099, Taif, 21944 Saudi Arabia; 8 Addiction and Neuroscience Research Unit, Health Science Campus, Taif University, P.O. Box 11099, Taif, 21944 Saudi Arabia

**Keywords:** High-throughput screening, Virtual drug screening, Cheminformatics, Computational chemistry

## Abstract

Human carbonic anhydrase XII (hCA XII) isozyme is of high therapeutic value as a pharmacological target and biomarker for different types of cancer. The hCA XII is one of the crucial effectors that regulates extracellular and intracellular pH and affects cancer cell proliferation, invasion, growth and metastasis. Despite the fact that interaction features of hCAs inhibitors with the catalytic site of the enzyme are well described, lack in the selectivity of the traditional hCA inhibitors based on the sulfonamide group or related motifs is an urgent issue. Moreover, drugs containing sulfanomides can cause sulfa allergies. Thus, identification of novel non-classical inhibitors of hCA XII is of high priority and is currently the subject of a vast field of study. This study was devoted to the identification of novel potential hCA XII inhibitors using comprehensive set of computational approaches for drug design discovery: generation and validation of structure- and ligand-based pharmacophore models, molecular docking, re-scoring of virtual screening results with MMGBSA, molecular dynamics simulations, etc. As the results of the study several compounds with alternative to classical inhibitors chemical scaffolds, in particular one of coumarins derivative, have been identified and are of high interest as potential non-classical hCA XII inhibitors.

## Introduction

Carbonic anhydrases (CAs, EC 4.2.1.1) are zinc metalloenzymes that catalyze the reversible hydration of carbon dioxide into bicarbonate and a proton^1^. Sixteen CA isoforms are found in humans and all vary in kinetic properties, subcellular localization and distribution to the tissues. Human carbonic anhydrase XII (hCA XII) is induced by hypoxia and highly expressed within the hypoxic core of many solid tumor types^2^. The hCA XII regulates extracellular and intracellular pH homeostasis of the cancer cells, thus, mediating cancer cells invasion, proliferation, metastasis, progression and tumor growth^3–5^.

In general, CA inhibitors (CAIs) are compounds equipped with an effective zinc-binding group (ZBG) capable of chelating the prosthetic zinc ion placed inside the hCA binding site that is essential for these enzymes' catalytic action. Sulfonamide moieties or related structural motifs represent the most common ZBGs shared by typical CAIs (such as sulfamides and sulfamates) and are the most significant groups of CAIs with multiple ligands with high inhibitory potency have been recorded to date^6^. These groups are especially effective in endowing high-affinity small-molecule ligands with hCAs, as they enable not only the proper coordination of the catalytic zinc ion, but also the forming of associations of H-bonds with key protein residues located in the zinc-binding cavity area. A great amount of effort has been put in drug design and discovery of hCA XII potential inhibitors^7–15^. Currently, SLC-011, one of the most promising compounds that is also based on the ureido-substituted benzene sulphonamide core (USB) has reached phase Ib/II of clinical trials as hCA IX/XII inhibitor^16,17^. However, because of the high amino acid conservation found at the level of their catalytic site and adjacent regions in the various hCA isoforms, most of these ligands are insufficiently selective against particular hCAs, including hCA XII which is currently the subject of a vast field of study^18^. In addition, a small but significant percentage of the general population cannot be treated with sulfonamide-based compounds due to a sulfa allergy^19,20^. Therefore, novel CAIs should be not only isoform specific, but also non-classical, i.e. not based on sulfonamides, sulfamates, or sulfamides^20^. Non-classical CAIs are an especially useful resource in this sense for finding isoform specificity and avoiding possible off-target events, side effects and adverse reactions (such as sulfur allergies) associated with the use of sulfurized ligands^20^. Notwithstanding, the development of hCA IX/XII selectivity inhibitors over hCA I/II, which are ubiquitously distributed and involved in key physiological processes, is still a difficult challenge, although some examples of selective ligands have been reported^21,22^.

Computer-aided drug design (CADD) comprises a broad range of theoretical and computational approaches that are part of modern drug discovery^23,24^. CADD methods have made key contributions to the development of drugs that are in clinical use or in clinical trials^25,26^. Such methods have emerged and evolved along with experimental approaches used in drug design. This study was devoted to identification of novel potential non-classical hCA XII inhibitors using the combination of computational approaches, including pharmacophore modeling (structure-, and ligand-based), molecular docking, MMGBSA re-scoring and molecular dynamics simulations.

## Materials and methods

**Figure 1:**
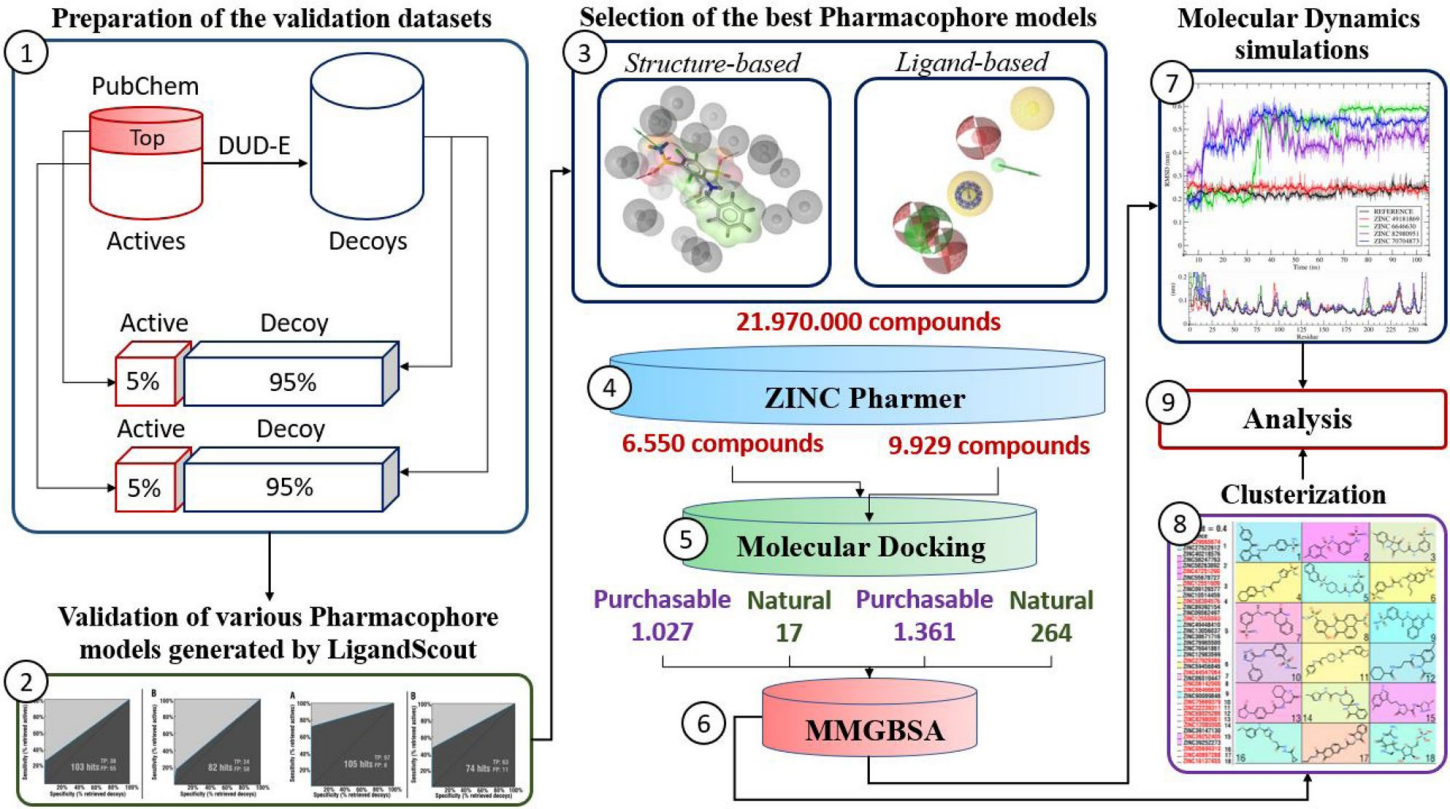
Schematic representation of general methodology and approaches applied in the study. Schematic illustration was drawn using Adobe Illustrator 2018 (www.adobe.com/products/illustrator).

Combination of the several computer-aided drug design and discovery methods, tools and approaches were applied in this study (Fig. 1). Structure- and ligand-based pharmacophores modelling was used for initial filtration of ZINC database of chemical compounds. Molecular docking and additional re-scoring with MMGBSA were used for further filtration and identification of potential hit compounds. Molecular dynamics simulations were used for assessment of interaction stability between hCA XII isozyme and top identified chemical compounds. Additionally, chemical scaffolds of identified compounds were analyzed and compared to the reference ligand (classical hCA inhibitor).

### Structure-based pharmacophore generation

Structure-based pharmacophore model was generated using LigandScout v4.4^27^. 22 crystal structures of the hcA XII in complexes with inhibitors (X-ray resolution < 2 Å) were downloaded from Protein Data Bank (PDB)^28^. Several approaches for the structure-based pharmacophore model generation were tested: (1) based on the separate crystal structures, (2) based on the combination of different crystal structures, (3) merged and (4) shared pharmacophores. Best pharmacophore model was generated using crystal structure of the catalytic domain of human carbonic anhydrase isozyme XII with benzene sulfonamide derivative (PDB ID: 4QJW, resolution 1.55 Å, chain C). All features that characterize interactions with the water molecules and hydrophobic interactions were removed from the obtained pharmacophore model. Additionally, exclusion volumes coat feature of LigandScout v4.4 was applied.

### Ligand-based pharmacophore generation

Ligand-based pharmacophore model was also generated using LigandScout v4.4^27^. 135 compounds active against hCA XII enzyme were downloaded from PubChem^29^ BioAssay dataset. These 135 active compounds were clusterized using pharmacophore alignment score as a similarity measure and the average method for cluster distance calculations. Maximum number of the conformations and cluster distance values were set to 3 and 0.4, respectively. As a result, 14 clusters were identified and representative compounds of these clusters were used for the creation of ligand-based pharmacophore models (Table S1). The final best ligand-based pharmacophore model was obtained based on the biggest cluster. “H bond donor” feature was removed from the selected ligand-based pharmacophore model.

### Validation of generated structure- and ligand-based pharmacophore models

2500 compounds with Ki < 10 against hCA XII enzyme were downloaded from PubChem BioAssay and ChEMBL^30^ databases. 5000 decoy compounds were generated using a Database of Useful Decoys: Enhanced (DUD-E)^31^ and were not included in the validation datasets in order to avoid bias. 3D structures of decoys were generated by Open Babel v3.1.1^32^ software based on the SMILES obtained by using DUD-E. Two separate validation datasets were prepared and each of them consisted of 5% active compounds and 95% decoys. First validation dataset included top 135 active compounds based on the Ki values. Second dataset included 135 randomly selected active compounds. Hydrogens were added to all compounds using Open Babel v3.1.1 software. The iConBest method of LigandsScout v4.4 was used for the generation of the conformations for active and decoy compounds.

For the validation of the generated pharmacophore models several widely established metrics were used. The area value under the receiver operating characteristic (ROC AUC) ranges from 0 to 1, where 1 is perfect classification, while values below 0.5 indicate random classification. ROC is widely used to evaluate virtual screening and pharmacophore modeling methods^33,34^ and defined as a graphical representation of the test sensitivity in relation to its specificity or false-positive rate. The AUC is the probability of active compounds being ranked earlier than decoy compounds. The classifier “precision” represents the share of true positives (TP) among all hits (TP/(TP + FP, where FP is false positive compounds)). The classifier “specificity” represents the ratio of the active compounds found in the list of compounds identified as “true positives” (TP/A, where A is active compounds). The “Specificity” classifier is the ratio of true negative compounds to all compounds in the database, excluding active ones. Enrichment Factor (EF) measures the fraction of active compounds found in a specific percentage, solving the problem of comparing the results for datasets with different active/inactive compound ratios^35^. The EF for 1, 5, 10 and 100% was calculated for the share of true positives among the molecules identified as hit compounds using generated pharmacophore models.

### Virtual screening

ZINC “purchasable” (21,777,093 compounds) and ZINC “natural and derivatives” (197, 488 compounds) datasets were used for the selection of library of compounds. ZINCPharmer^36^ was used for the filtration of these datasets using generated structure-based and ligand-based pharmacophore models. Identified hit compounds were used for the molecular docking against the active site of the hCA XII enzyme (PDB ID 4QJW). AutoDock Tools^37^ was used for the estimation of the grid box (< 27 Å), calculation of the protein’s and compounds’ charges and the addition of the polar hydrogens. Virtual screening was performed using AutoDock Vina software^38^, which has been regarded as highly efficient software for molecular docking and virtual screening procedures based on the recent benchmark studies among both academic and commercial software^39,40^. AutoDock Vina uses “Iterated Local Search global optimizer” similar to that by Abagyan et al. and Broyden-Fletcher-Goldfarb-Shanno (BFGS), which is quasi-Newton method for the local optimization, as a search algorithm and hybrid scoring function (empirical + knowledge-based function) inspired in the X-Score function^38^. Standard, recommended by the developers, parameters were used for the virtual screening procedure.

### Re-scoring using MMGBSA method

The algorithm for the molecular mechanics-generalized Born surface area (MMGBSA) calculations is based on a freely-available AmberTools suite. The algorithm can be described in three stages, (1) receptor and ligands parametrization, (2) minimization and (3) MM/GBSA and MM/PBSA calculations. At the first stage, the ff14SB force field^41^ is used to describe protein parameters, and General Amber Force Field (GAFF)^42^ with AM1-BCC charge model^43^ is used for small molecule parametrization. Next, the algorithm prepares necessary input files (coordinates and topologies) with mbondi3 radii using LEaP. Minimization is performed in generalized Born implicit solvent models (igb = 8) using the sander engine. Finally, for the free energy calculations, the algorithm uses the MMPBSA.py program^44^ for the MMGBSA calculations. The algorithm^45^ is implemented in a bash script and can be run in parallel in most Linux distributions without any additional libraries for parallelization. The full code is available at the following link: https://github.com/sahakyanhk/iPBSA.

### Molecular dynamics simulations

The molecular dynamics simulations were carried out using AMBER20^46^ molecular dynamics package. The ff14SB force field was used for protein parametrization and GAFF for the ligand parameterization with AM1-BCC charge model. Minimized conformations of complexes of hCA XII with bound compounds were taken from the previous step (re-scoring of docked complexes with MMGBSA) and used as starting positions for corresponding simulations. The complexes were solvated in TIP3P water model and Na^+^/Cl^−^ ions at 150 mM concentration^47^. The Monte Carlo barostat^48^ with reference pressure at 1 bar and Langevin thermostat^49^ with collision frequency (gamma_ln) 2 ps^−1^ were used to keep the temperature at 310.15 K. The Particle Mesh Ewald (PME) method with 1.0 nm cutoff was used for the long-range electrostatic interactions. Bonds involving hydrogen were constrained using the SHAKE algorithm with 2 fs integration step^50^. Each simulation consisted of 5 ns of system minimization and equilibration and 100 ns of conventional molecular dynamics simulation. Finally, for every simulation, binding free energies were re-calculated using the same MMGBSA method and MMPBSA.py program, using 250 snapshots with equal intervals collected from the last 20 ns of simulation. RMSD and SASA was calculated as indicators of stability of studied complexes during simulation. Besides, RMSF analysis was performed to measure the average atomic flexibility of the Cα atoms of the docked complexes. Radius of gyration was calculated as an indicator of protein structure compactness during simulation. Hydrogen bonding analysis was performed to identify similarities and differences in interaction patterns between studied compounds and amino acid residues of hCA XII binding site.

## Results and discussion

### Ligand-based pharmacophore generation

**Figure 2:**
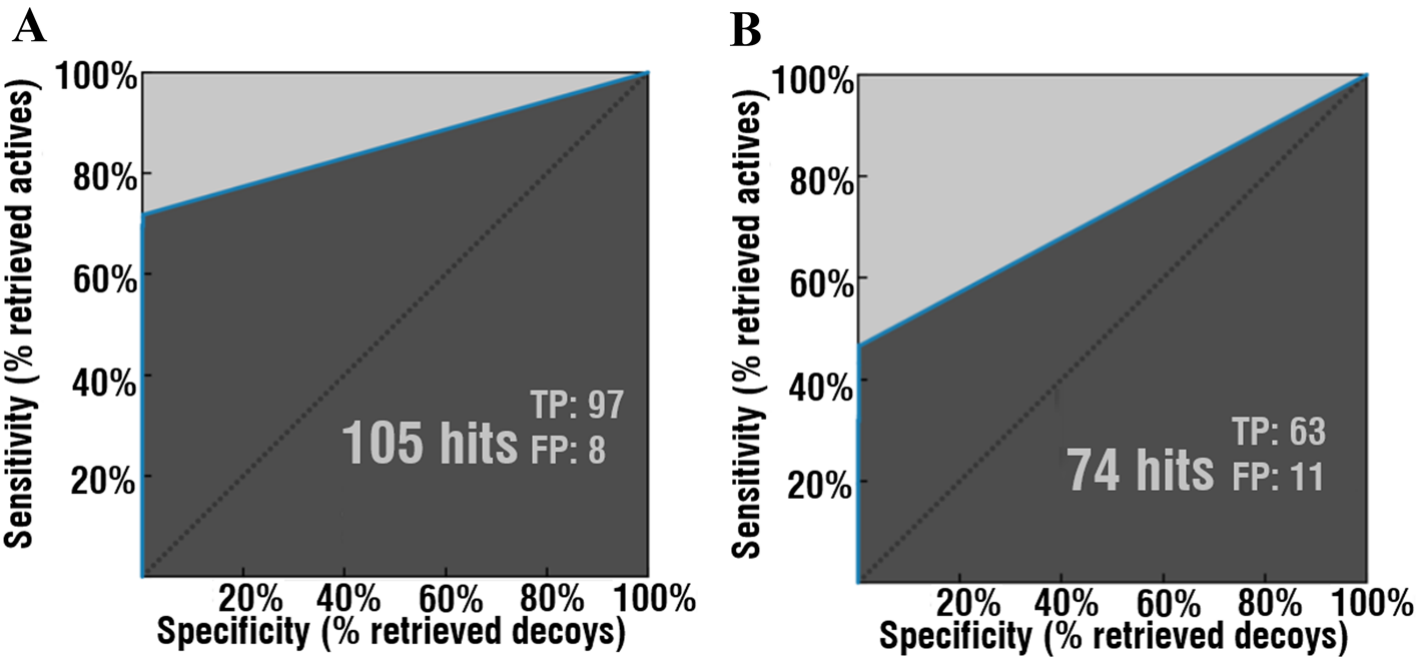
Validation of the generated ligand-based pharmacophore on the two datasets (**A**,**B**) Identified hit compounds out of 2635 total compounds (135 actives, 2500 decoys). Figure was obtained using LigandScout v4.4^27^ (www.inteligand.com/ligandscout/). Schematic illustration was drawn using Adobe Illustrator 2018 (www.adobe.com/products/illustrator).

As a result of the clusterization of 135 compounds active against hCA XII, 14 clusters have been obtained. The best ligand-based pharmacophore model was obtained based on the biggest cluster (Supplementary Figure S1). The final ligand-based pharmacophore model included three “H-bond (acceptor)” features, two “H-bond (donor)” features, one “hydrophobic” and “aromatic” features of the LigandScout v4.4. Validation tests of the generated ligand-based pharmacophore model demonstrated high results on two separate datasets. First dataset (Fig. 2A): 105 hits (97 TP, 8 FP), AUC 1, 5, 10, 100−1, 1, 1, 0.86; EF 1, 5, 10, 100−19.8, 18.3, 18.3, 18.3. Second dataset (Fig. 2B): 74 hit compounds (63 TP, 11 FP) AUC 1, 5, 10, 100−1, 1, 1, 0.73; EF 1, 5, 10, 100−19.8, 16.9, 16.9, 16.9.

### Structure-based pharmacophore generation

**Figure 3:**
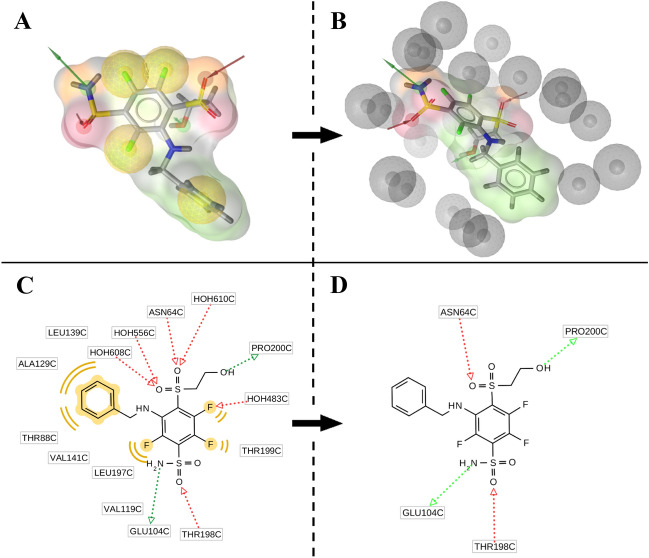
The best generated structure-based pharmacophore model. 3D (**A**) and 2D (**C**) representations of the pharmacophore model with all initial features. Final pharmacophore models after removing features responsible for the interactions with water molecules and hydrophobic interactions (**B**,**D**) and addition of the exclusion volumes coat (**B**). Figures of structure-based pharmacophore model was obtained using LigandScout v4.4^27^ (www.inteligand.com/ligandscout/). Schematic illustration was drawn using Adobe Illustrator 2018 (www.adobe.com/products/illustrator).

Structure-based pharmacophore model generated based on the crystal structure of catalytic domain of hCA isozyme XII with benzenesulfonamide derivative (PDB ID: 4QJW) demonstrated LigandScout’s binding affinity score of − 17.18, which includes interaction and desolvation energies. The selected structure-based pharmacophore model included two “H-bond (acceptor)” features that are important for the interaction of the ligand with ASN64 and THR198 of the active site of hCA XII enzyme, and two “H-bond (donor) feature that are important for interaction with PRO200 and GLU104 (Fig. 3).

**Figure 4:**
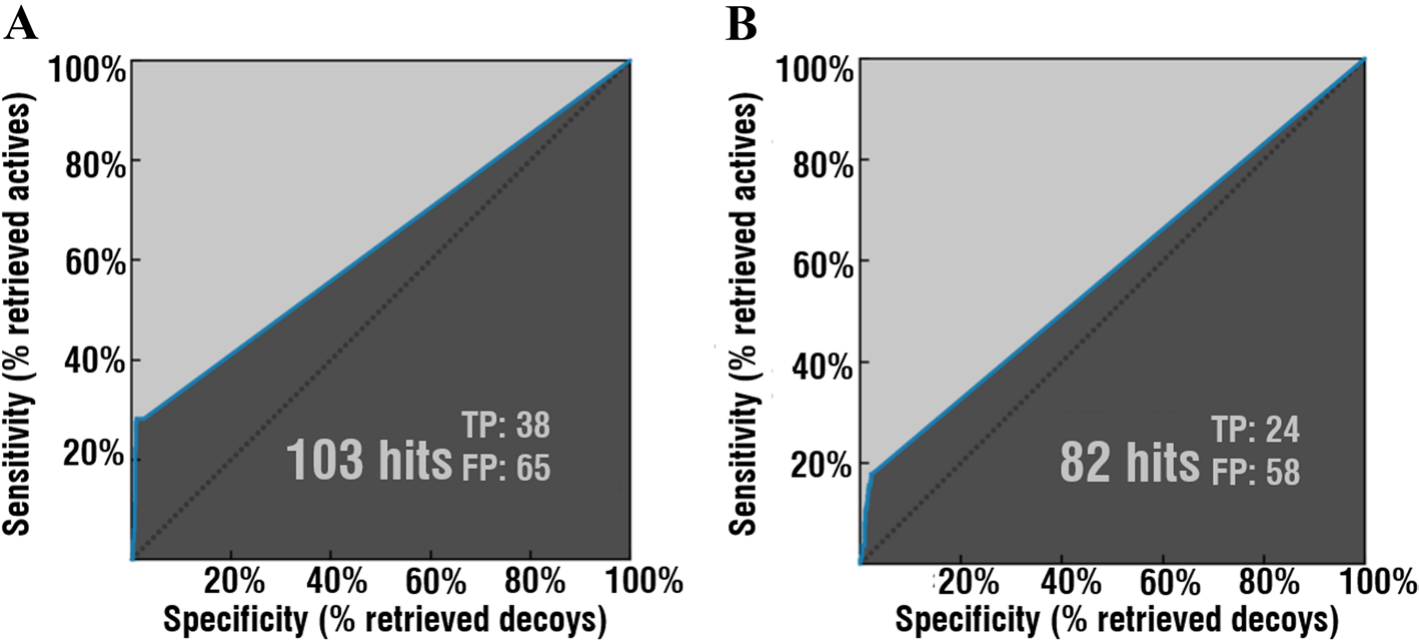
Validation of the generated structure-based pharmacophore on the two datasets (**A**,**B**). Identified hit compounds out of 2635 total compounds (135 actives, 2500 decoys). Figure was obtained using LigandScout v4.4^27^ (www.inteligand.com/ligandscout/). Schematic illustration was drawn using Adobe Illustrator 2018 (www.adobe.com/products/illustrator).

Validation of structure-based pharmacophore model in the case of the first dataset (Fig. 4A) demonstrated following values: 103 hit compounds (38 TP, 65 FP), AUC 1, 5, 10, 100−0.72, 0.93, 0.95, 0.63; EF 1, 5, 10, 100−7.6, 7.3, 7.3, 7.3. In the case of the second validation dataset (Fig. 4B): 82 hit compounds (24 TP, 58 FP), AUC 1, 5, 10, 100−0.97, 0.92, 0.93, 0.58; EF 1, 5, 10, 100−3.8, 5.8, 5.8, 5.8 (Fig. 4).

### Filtration of ZINC database using generated pharmacophore models

Both selected ligand-based and structure-based pharmacophore models were applied to filter ZINC “purchasable” and “natural and derivatives” compounds datasets (total of 21.970.000 compounds) using ZINCPharmer, separately. 6357 and 193 hit compounds were identified from “purchasable” and “natural and derivatives” datasets, respectively, using a structure-based pharmacophore model. Other 8415 and 1514 hit compounds were identified using a ligand-based pharmacophore model. All identified hit compounds were used for molecular docking against the active site of the hCA XII enzyme.

### Molecular docking

1361 “purchasable” and 264 “natural and derivatives” compounds with similar or higher docking scores compared to the reference molecule (co-crystallized ligand of 4QJW, benzenesulfonamide derivative: − 8 kcal/mol) were identified as the result of molecular docking of hit compounds obtained by application of the ligand-based pharmacophore (Table S2). In case of hit compounds identified by structure-based pharmacophore, 1027 “purchasable” and 17 “natural and derivatives” compounds were identified as a result of molecular docking (Table S3). All of the compounds with similar or higher docking scores in comparison to the reference ligand were passed to the additional stages of minimization and re-scoring using MMGBSA method.

### MMGBSA re-scoring

Only 38 compounds from the “purchasable” (Table 1) and 2 from “natural and derivatives” (Table 2) datasets demonstrated similar or higher binding energy values than reference ligand as the result of MMGBSA re-scoring. 24 out of aforementioned 38 compounds were obtained with the use of structure-based pharmacophore model and the rest 15 with the use of the ligand-based pharmacophore model. Both compounds identified from “natural and derivatives” dataset were obtained with the use of a ligand-based pharmacophore model.

**Table 1:** Binding energies of the top “purchasable” compounds after re-scoring using MM-GBSA.

Structure-based approach				Ligand-based approach			
ZINC ID	Energy (kJ/mol)	ZINC ID	Energy (kJ/mol)	ZINC ID	Energy (kJ/mol)	ZINC ID	Energy (kJ/mol)
ZINC66466630	− 157,83	ZINC76941861	− 129,56	ZINC82980951	− 159,25	ZINC21761334	− 133,43
ZINC16137455	− 153,97	ZINC39147130	− 128,51	ZINC68025286	− 147,68	ZINC10514459	− 130,47
ZINC90089846	− 152,20	ZINC75669379	− 127,26	ZINC27522612	− 144,36	ZINC38671716	− 129,54
ZINC76965500	− 146,49	ZINC39252273	− 127,24	ZINC12555593	− 141,68	ZINC55678727	− 128,52
ZINC05699310	− 139,63	ZINC12551609	− 126,40	ZINC29565674	− 139,19	ZINC27929386	− 128,16
ZINC09126577	− 139,14	ZINC89392154	− 126,25	ZINC59456846	− 137,50	ZINC13056037	− 127,18
ZINC44547064	− 138,05	ZINC12085595	− 125,75	ZINC49448410	− 136,54	ZINC40897288	− 124,45
ZINC09562497	− 135,91	ZINC39252405	− 125,39	ZINC06510447	− 136,40	ligand_4qjw	− 124,20
ZINC22239311	− 134,62	ZINC12983599	− 124,99				
ZINC47251290	− 134,41	ZINC58304576	− 124,67				
ZINC58247763	− 132,89	ZINC06142500	− 124,23				
ZINC40218576	− 131,14	ligand_4qjw	− 124,20				
ZINC58263892	− 129,77

**Table 2:** Binding energies of the top “natural and derivatives” compounds after re-scoring using MMGBSA.

Structure-based approach		Ligand-based approach	
ZINC ID	Energy (kJ/mol)	ZINC ID	Energy (kJ/mol)
ZINC70704873	− 105,68	ZINC49181869	− 139,62
ZINC04221765	− 93,03	ZINC49181861	− 133,30
ZINC02131655	− 85,34	ZINC49181866	− 120,74
ZINC70699917	− 77,17	ZINC08829478	− 111,70
ZINC15958674	− 76,88	ZINC08792367	− 109,86

**Figure 5:**
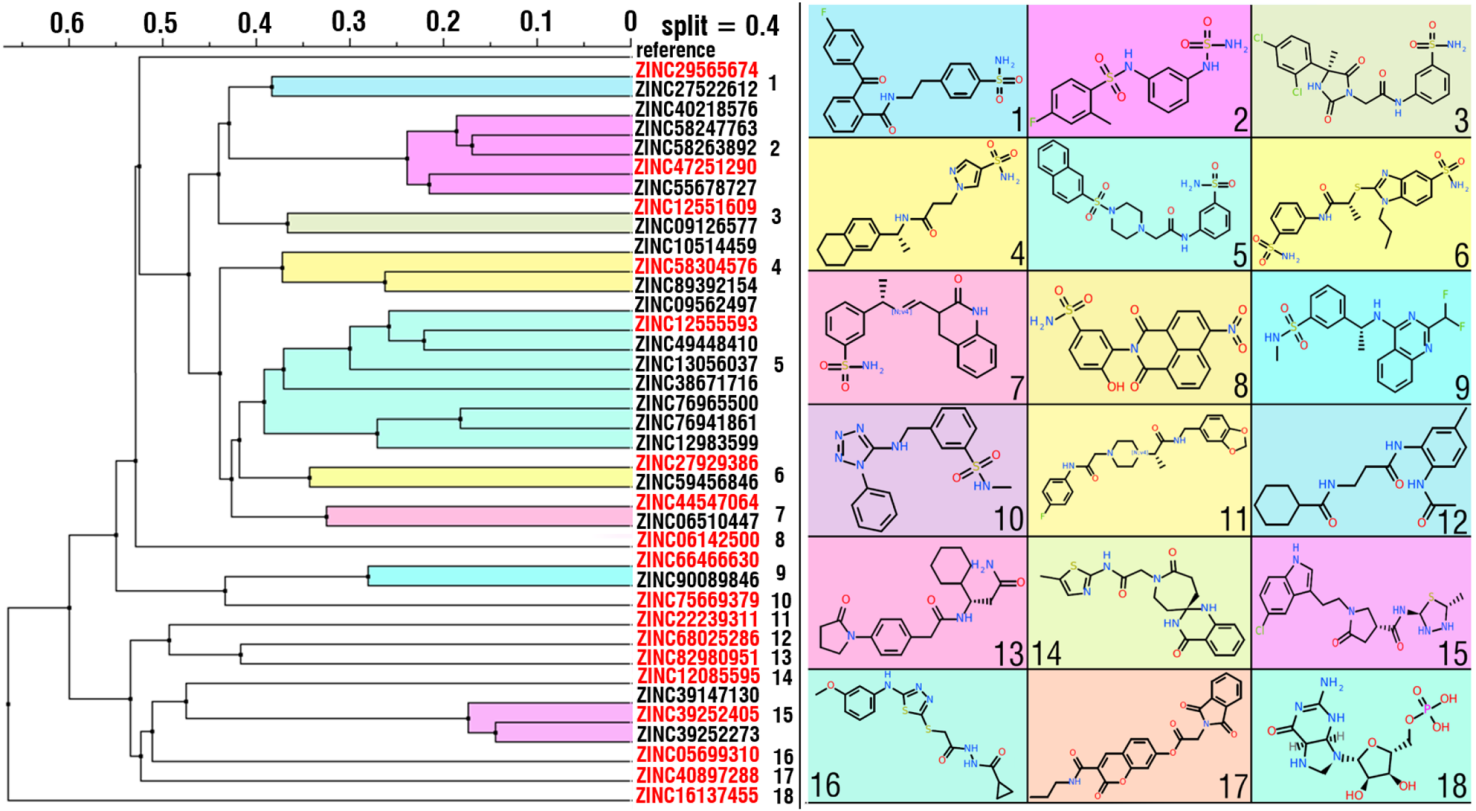
Results of the clusterization of the identified compounds as the result of MMGBSA re-scoring procedure. 2D structures of representative are presented for all 18 clusters. IDs of representative compounds of clusters with more than one compound are colored in red. Clusterization dendrogram and figures of chemical structures were obtained using ICM-PRO^51^ (http://www.molsoft.com/icm_pro.html). Schematic illustration was drawn using Adobe Illustrator 2018 (www.adobe.com/products/illustrator).

In order to analyze chemical diversity of identified compounds, an agglomerative hierarchical clustering method based on the “ECFP” fingerprint implemented in ICM-Pro software^51^ was performed (Fig. 5). As a result of the clusterization of the identified 38 compounds, 18 clusters were obtained. As expected, most of the identified compounds (Fig. 5, clusters 1–10) have sulfonamide moieties or related structural motifs that are widely known to promote small-molecules interaction with hCAs active site. However, several of the identified compounds (Fig. 5, clusters 11–18) have different from sulfonamide chemical moieties. These compounds include cyclohexanecaboxiamide, propanamide, acetamide, cyclopropanecarbohydrazide, thiadiazolidine, cyclopropane, carbohydrazide, chromen-7, tetrahydrofuran derivatives and analogues.

Remarkably, one of these compounds, identified with the use of ligand-based pharmacophore (ZINC82980951), showed the lowest binding energy among all compounds as the result of MMGBSA re-scoring (Table 1). Compounds that are chemically different from the traditional hCA XII inhibitors (which include sulfonamide chemical group) are of special interest for further analysis and investigation as potential alternative hCA XII inhibitors.

In the case of the “natural and derivatives” compounds only two compounds (ZINC49181869 and ZINC49181861, Table 2), which are stereoisomers of the same coumarin derivative, showed higher binding energies than reference ligand.

### Molecular dynamics

From the list of compounds, identified from the “purchasable” dataset (Table 1), two compounds with lowest binding energy (one identified using structure-based pharmacophore—ZINC66466630 and another one using ligand-based pharmacophore model—ZINC82980951) were selected for additional MD simulations. In the case of the “natural and derivatives” dataset (Table 2), two compounds with the lowest binding energies, identified using structure-based (ZINC70704873) and ligand-based pharmacophore (ZINC49181869) models, were also selected for MD simulations.

**Figure 6:**
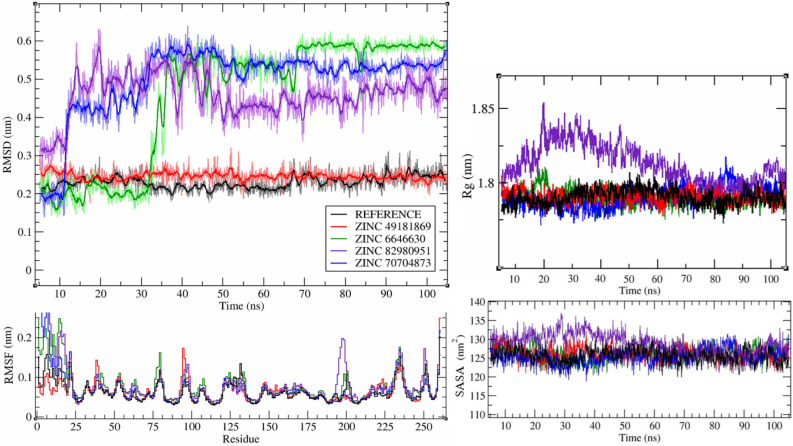
RMSD values of studied compounds and Rg, RMSF and SASA values of hCA XII during performed MD simulations. First 5 ns represent equilibration stage of performed MD simulations.

The reference compound (co-crystallized ligand of 4QJW structure) was stable during the whole simulation and has maintained conformation close to its initial crystal state (fluctuations around 0.05 nm, Fig. 6).

From the four tested compounds only ZINC49181869 was stable during the whole simulation and also maintained conformation close to the one predicted by molecular docking fluctuations around 0.05 nm. Compound ZINC70704873 stabilized after ~ 55 ns of the simulation and maintains its conformation with fluctuations around ~ 0.05 nm. ZINC66466630 stabilized after ~ 70 ns and maintains its conformation with fluctuations around ~ 0.025 nm. ZINC82980951 stabilized after ~ 50 ns and maintains its conformation with fluctuations around ~ 0.1 nm. Based on the obtained RMSD, RMSF, Rg and SASA values all studied complexes stabilize within performed simulations.

**Figure 7:**
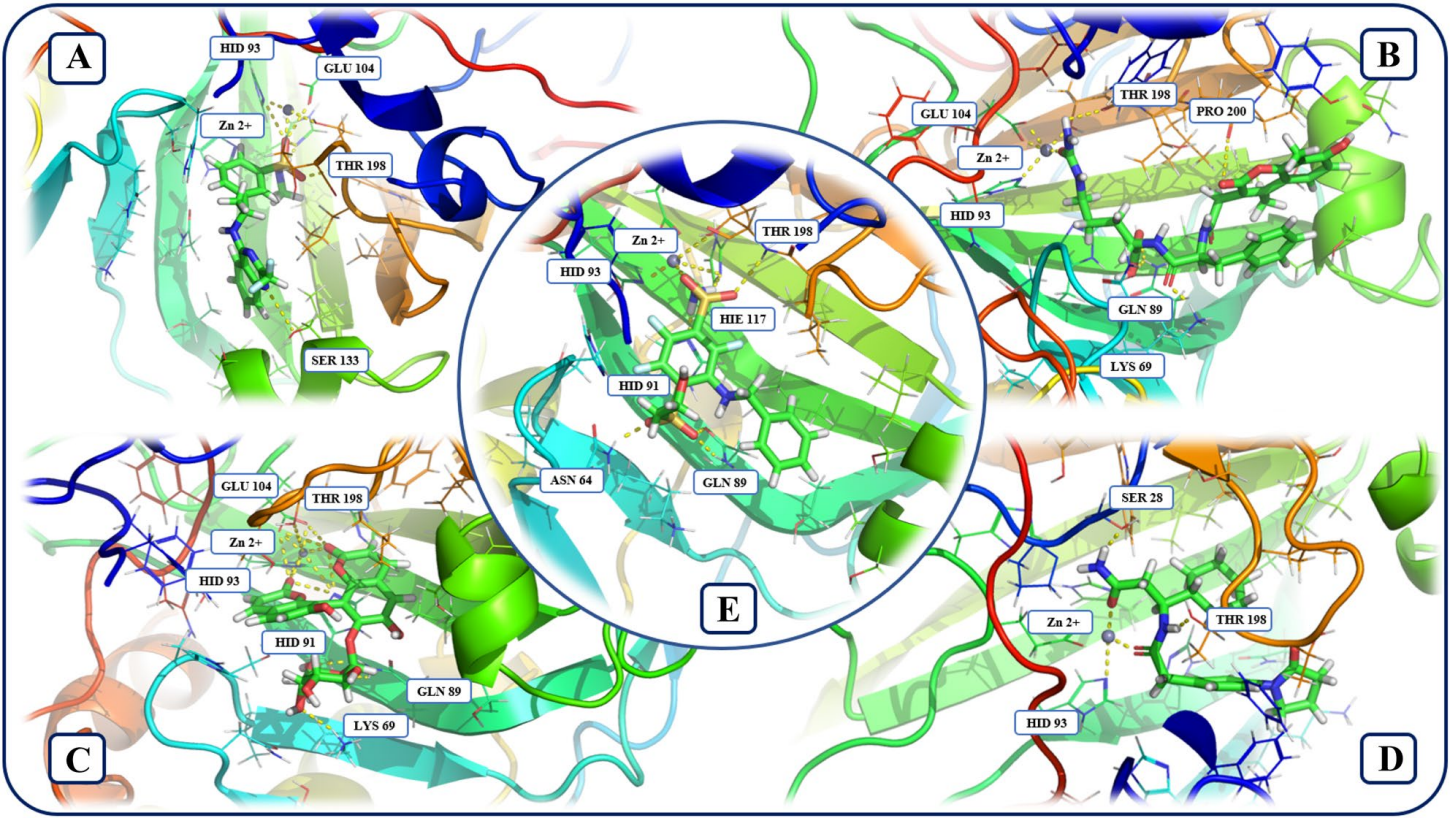
Interaction of the selected compounds with amino acid residues and Zn^2+^ ion in the binding site of the hCA XII isozyme. (**A**) ZINC66466630, (**B**) ZINC70704873, (**C**) ZINC49181869, (**D**) ZINC82980951, (**E**) Reference. Figures of complexes were obtained using PyMOL v. 2.3.2 (https://pymol.org). Schematic illustration was drawn using Adobe Illustrator 2018 (www.adobe.com/products/illustrator).

In its stable conformation, during molecular dynamics simulation, the reference ligand had five hydrogen bonds with the following amino acid residues of active site of the hCA XII enzyme: HIE 117, HID 91, GLN 89, ASN 64, THR 198. In the presence of the reference ligand, HID 93, HIE 117 and THR 198 amino acid residues coordinate Zn^2+^ ion (Fig. 7).

Similarities and differences in interaction patterns of the amino acid residues of hCA XII binding site with compounds and reference ligand is of particular interest for the evaluation of the studied compounds as potential alternative inhibitors of the hCA XII. ZINC66466630 and ZINC82980951 compounds and reference ligand have hydrogen bond with the THR 198 amino acid residue of the hCA XII active site. ZINC70704873, as the reference ligand, has hydrogen bonds with THR 198 and GLN 89 residues. ZINC49181869 and reference ligand both have hydrogen bonds with GLN 89 and HID 91 residues.

At the same time, all studied compounds have unique hydrogen bonds with other amino acid residues of the hCA XII isozyme. ZINC66466630 has hydrogen bonds with HID 93, GLU 104 and SER 133 residues. ZINC70704873 has hydrogen bonds with PRO 200 and LYS 69. ZINC49181869 has hydrogen bonds with HID 93, GLU 104 and LYS 71 residues. Finally, ZINC82980951 has hydrogen bond with SER 30 amino acid residue.

Information on the involvement of amino acid residues of the hCA XII binding site in coordination of Zn^2+^ ion in the presence of different compounds is also of high value for drug design and discovery of potential hCA inhibitors. Common amino acid residues that interact with the Zn^2+^ in the presence of tested compounds and reference ligand: HID 93, THR 198 (ZINC66466630, ZINC70704873, ZINC49181869), HID 93 (ZINC82980951). Unique, in comparison to the reference ligand, amino acid residues that interact with the Zn in the presence of the tested compounds: GLU 104 (ZINC70704873), HID 91 and GLU 104 (ZINC49181869), HID 93 (ZINC82980951).

**Figure 8:**
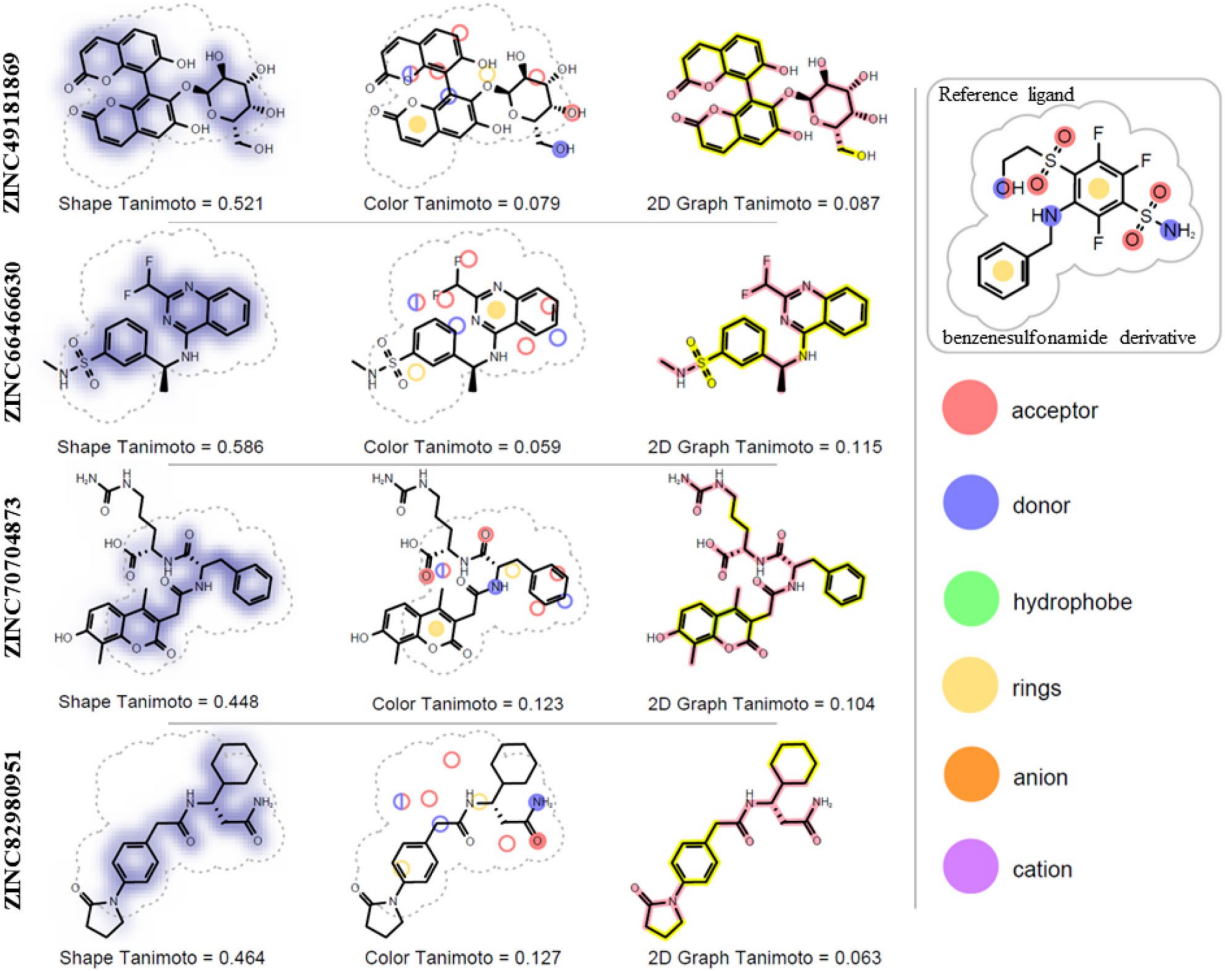
Comparison of the shape and chemical structures of identified compounds with the benzensulfonamide derivative (representative classical inhibitor of hCA XII). Figure was obtained using OpenEye ROCS’s^52^ ROCSReport utility (https://www.eyesopen.com/rocs).

Additionally, comparison of the shapes and chemical structures of the selected compounds to the reference ligand (classical inhibitor of classical inhibitor of hCA XII) was performed using ROCS_report utility tool (Fig. 8).

Only one out of four studied compounds (ZINC66466630) has sulfonamide group, which is inherent in classical hCA inhibitors. Three other studied compounds have alternative to classical inhibitors chemical structures.

One of these compounds—ZINC49181869 (coumarin derivative) is of higher interest as potential alternative inhibitor of the hCA XII, since it demonstrated relatively good parameters based on various indicators. This compound showed exceptional stability during the whole length of molecular dynamics simulation. It also has high affinity to the hCA XII isozyme (− 139,62 kJ/mol) based on the MMGBSA calculations. Besides, it is a natural compound that has various good ADMET properties predicted by SwissADME^53^ (consensus Log P of 0.58; Log S of around − 3.5 and is not inhibitor of CYP1A2, CYP2C19, CYP2C9, CYP2D6 and CYP3A4). Remarkably coumarins are regarded as a promising new class of non-classical inhibitors of CAs^20,54^.

## Conclusion

Despite the fact that interaction features of hCAs inhibitors with the catalytic site of the hCA enzyme are well described, classical inhibitors that have sulfonamides group or related motifs are lacking selectivity to the particular hCA isoforms and can also cause sulfa allergies in patients. Novel inhibitors of hCA XII should be both, isoform specific and non-classical, i.e. not based on sulfonamides, sulfamates, or sulfamides. This study was devoted to the identification of novel potential hCA XII inhibitors using comprehensive set of computational approaches for drug design discovery. As the results of the study several compounds with alternative to classical inhibitors chemical scaffolds have been identified and are of high interest as potential non-classical hCA XII inhibitors. The most promising out of identified compounds is coumarin derivative (ZINC49181869) that demonstrated relatively better indicators and properties based on the performed calculations and analysis. Remarkably, coumarin derivatives were recognized as a new class of non-classical inhibitors of CAs based on several relatively recent studies. The results of our study signify the potency of coumarins as non-classical inhibitor of hCA XII isozyme.

## Supplementary Information

Supplementary Table S1

Supplementary Table S2

Supplementary Table S4

Supplementary Figure S1
