# Supplementary material for: Identification of non-classical hCA XII inhibitors using combination of computational approaches for drug design and discovery
Source: Sci Rep. 2021 Jul 30;11:15516. doi: 10.1038/s41598-021-94809-x (PMC8324906; doi:10.1038/s41598-021-94809-x)
Supplement: Supplementary file 4 — Supplementary Figure S1 [file 41598_2021_94809_MOESM4_ESM.pdf]

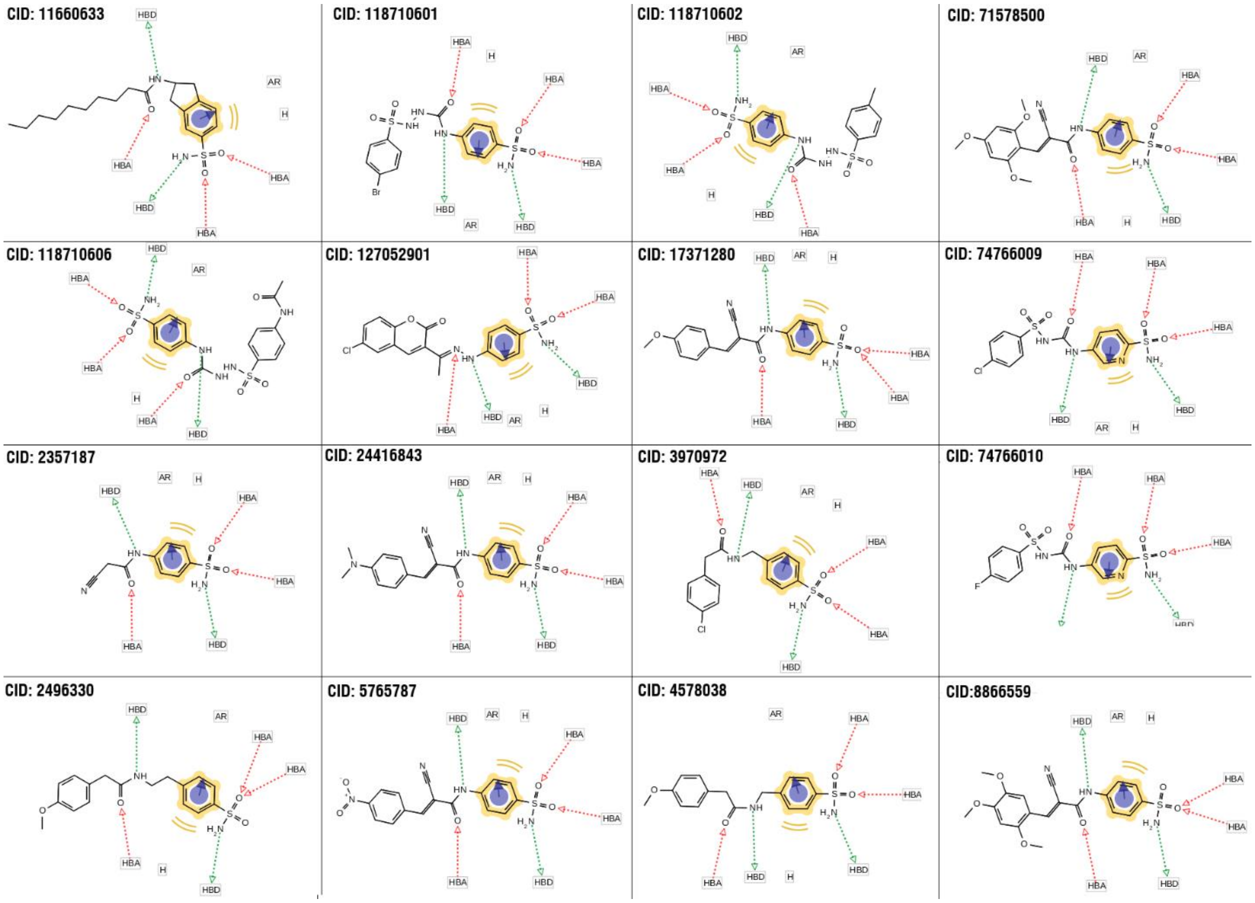

Supplementary Figure S1. Active compounds (with PubChem CIDs) of selected cluster used for the generation of ligand-based pharmacophore model. Figure was obtained using LigandScout v4.4[27] (www.inteligand.com/ligandscout/).
